# Long-range fine particulate matter from the 2002 Quebec forest fires and daily mortality in Greater Boston and New York City

**DOI:** 10.1007/s11869-015-0332-9

**Published:** 2015-02-28

**Authors:** Ke Zu, Ge Tao, Christopher Long, Julie Goodman, Peter Valberg

**Affiliations:** Gradient, 20 University Road, Cambridge, MA 02138 USA

**Keywords:** Air pollution, Wildfires, Fine particulate matter, PM_2.5_, Mortality, Natural experiment

## Abstract

During July 2002, forest fires in Quebec, Canada, blanketed the US East Coast with a plume of wood smoke. This “natural experiment” exposed large populations in northeastern US cities to significantly elevated concentrations of fine particulate matter (PM_2.5_), providing a unique opportunity to test the association between daily mortality and ambient PM_2.5_ levels that are uncorrelated with societal activity rhythms. We obtained PM_2.5_ measurement data and mortality data for a 4-week period in July 2002 for the Greater Boston metropolitan area (which has a population of over 1.7 million people) and New York City (which has a population of over 8 million people). Daily average PM_2.5_ concentrations were markedly increased for 3 days over this period, reaching as high as 63 μg/m^3^ for Greater Boston and 86 μg/m^3^ for New York City from background ambient levels of 4–48 μg/m^3^ in the non-smoke days. We examined temporal patterns of natural-cause deaths and 24-h ambient PM_2.5_ concentrations in July 2002 and did not observe any discernible increase in daily mortality subsequent to the dramatic elevation in ambient PM_2.5_ levels. Comparison to mortality rates over the same time periods in 2001 and 2003 showed no evidence of impact. Results from Poisson regression analyses suggest that 24-h ambient PM_2.5_ concentrations were not associated with daily mortality. In conclusion, substantial short-term elevation in PM_2.5_ concentrations from forest fire smoke were not followed by increased daily mortality in Greater Boston or New York City.

**Electronic supplementary material:**

The online version of this article (doi:10.1007/s11869-015-0332-9) contains supplementary material, which is available to authorized users.

## Background

A number of single-city and multicity time-series studies have evaluated the association between short-term population exposure to ambient fine particulate matter (PM_2.5_) and day-by-day mortality, many of which have reported small positive risk increments (US EPA 2009). For example, the National Morbidity, Mortality, and Air Pollution Study (NMMAPS), a large-scale collaborative project that aims to evaluate the health effects of ambient air pollution, conducted a national analysis of PM_2.5_ and mortality from 1999 to 2000 in 96 US cities and reported that a 10-μg/m^3^ increase in ambient PM_2.5_ was associated with a 0.29 % increase (95 % posterior interval 0.01, 0.57) in daily mortality at lag 1 (Dominici et al. 2007). Franklin et al. (2007) examined the relationship between ambient PM_2.5_ and daily mortality from 1997 to 2002 in 27 US cities and reported increases in all-cause mortality of 0.67 % (95 % confidence interval (CI) −0.12, 1.46), 1.21 % (95 % CI 0.29, 2.14), and 0.82 % (95 % CI 0.02, 1.63) for a 10-μg/m^3^ increase in PM_2.5_ concentrations at lag 0, lag 1, and lag 0–1, respectively. Zanobetti and Schwartz (2009) conducted a national analysis of ambient PM_2.5_-mortality associations from 1999 to 2005 in 112 US cities and reported a pooled estimate of 0.98 % (95 % CI 0.75, 1.22) increase in all-cause daily mortality for a 10-μg/m^3^ increase in PM_2.5_ at lag 0–1. A multicity study in Canada also reported statistically significant increases in daily mortality associated with increases in PM_2.5_ at lag 0 or lag 1, and the statistically significant risk estimates for PM_2.5_ at lag 1 persisted with adjustments for a second gaseous co-pollutant including ozone, nitrogen dioxide, carbon monoxide, and sulfur dioxide (Burnett et al. 2000). Based on the epidemiology evidence, including these studies, the United States Environmental Protection Agency (US EPA) concluded that there is a causal relationship between short-term exposures to PM_2.5_ and daily mortality (US EPA 2009).

Despite US EPA’s conclusion, uncertainty remains with regard to the causal interpretations of the observed associations between PM_2.5_ and mortality (Valberg 2004; Cox 2013). Societal activity elevates all types of combustion emissions, hence ambient air pollution levels, including PM_2.5_. The intensity of societal activity also correlates with stress, which is associated with mortality (Juth et al. 2008; Phillips et al. 1999, 2001, 2004; Smyth et al. 1999). In an analysis of correlations between heart attack risk and subjects’ daily activities, Peters et al. (2004) reported a role for “exposure-to-traffic” stress in heart attack risk but not for pollutant concentrations per se.

**Fig. 1:**
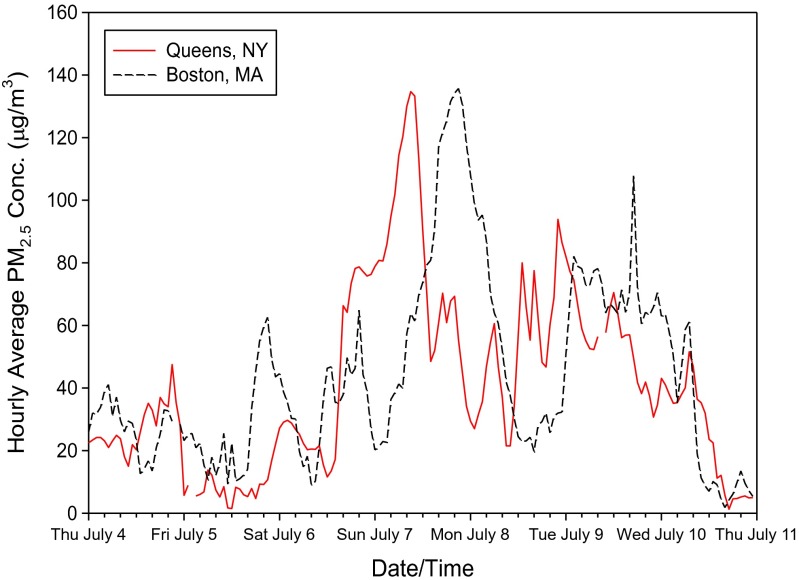
Hourly average PM_2.5_ concentrations for representative monitors in Greater Boston (Site ID 25-025-0043) and New York City (Site ID 36-081-0116) at the time of the air quality impacts from the July 2002 Quebec wildfires

To help address uncertainty regarding the effect of PM_2.5_, “natural experiments” may provide key evidence for causal determinations (Dominici et al. 2014). Smoke plumes from wild forest fires can travel over a long range, and satellite imagery can be used to track the smoke plumes and help identify potentially impacted populations (Chung and Le 1984; Chung and Kim 2008). In early July 2002, massive forest fires broke out in Quebec, Canada, leading to a smoke plume blanketing the US East Coast. Consequently, for several days, PM_2.5_ concentrations were markedly elevated in a number of major downwind cities in New England, New York, and the mid-Atlantic states. For example, the annual average PM_2.5_ concentration in 2002 for Boston was 15.0 μg/m^3^ (http://www.mass.gov/eea/docs/dep/air/02airrpt.pdf), but hourly measurements of PM_2.5_ concentrations in Boston on July 8, 2002, often exceeded 100 μg/m^3^ (Fig. 1). Air monitoring in Baltimore showed that 24-h average PM_2.5_ levels outdoors reached 86 μg/m^3^, and short-term outdoor levels approached 200 μg/m^3^ (Sapkota et al. 2005).

In our analysis, we examined the association between PM_2.5_ and mortality in Greater Boston and New York City during and after this natural experiment, wherein substantial short-term increases in PM_2.5_ levels were uncorrelated with local human activities.

## Materials and methods

### Study periods

Multiple forest fires occurred in Quebec, Canada, in early July 2002, leading to marked elevations in PM_2.5_ concentrations in urban areas along the US eastern seaboard, including Greater Boston and New York City (~800 to 1000 miles downwind of the fires). Thus, we evaluated PM_2.5_ and mortality from July 1–28, 2002, in these two large metropolitan areas. We hypothesized that the potential effect of short-term elevated PM_2.5_ on daily mortality would be observed within 1 week of smoke plume impact. Therefore, we defined July 7–16, 2002, as the wildfire-smoke-impacted days and the rest of July 2002 as the non-impacted days. We also evaluated two additional 4-week periods, matched on day of the week, in 2001 (July 2–29) and 2003 (June 30–July 27), to determine whether the observed daily mortality rates in 2002 differed from those for the matched time periods in 2001 and 2003.

### Mortality data

Daily mortality data for Greater Boston and New York City from 2001 to 2003 were obtained from the Massachusetts Department of Public Health and the New York City Department of Health and Mental Hygiene, respectively. Daily counts for total mortality (by natural causes), cardiovascular mortality, and respiratory mortality were calculated for July 1–28, 2002, and the matched periods in 2001 and 2003.

This study was approved by the Chesapeake IRB (Columbia, MD).

### Air pollution and meteorological data

Ambient PM_2.5_ data were obtained from the US EPA Air Quality System (AQS) website (http://www.epa.gov/ttn/airs/airsaqs/) for central-site monitoring locations in the Greater Boston area and each of the five New York City boroughs (Manhattan, Brooklyn, Queens, the Bronx, and Staten Island). PM_2.5_ data were available for five to six locations in the Greater Boston area, six to ten locations in Manhattan, three to five locations in Brooklyn, two to five locations in Queens, five locations in the Bronx, and four locations in Staten Island (the number of monitoring stations varied across the 3 years). Data availability differed across monitoring stations, with some stations having hourly data, some having daily data for each calendar day, and others having daily data for every third calendar day. The measurements of 24-h PM_2.5_ concentrations were well correlated between monitors in each city (correlation coefficients >0.8 in Greater Boston and >0.9 in New York City). Using both the available hourly and daily PM_2.5_ data, we calculated average 24-h PM_2.5_ concentrations across all monitors within the Greater Boston area and each New York City borough for the 4-week period in July 2002 and the matched by day-of-week time periods in 2001 and 2003.

Daily average measurements of meteorological factors, including ambient temperature and dew point temperature, were obtained from the National Climatic Data Center (NCDC) website (http://www.ncdc.noaa.gov/) for Boston’s Logan International Airport and New York City’s LaGuardia Airport. Daily average data on ambient and dew point temperatures were used to calculate values of apparent temperature, which is a metric for describing the perception of the combination of temperature and humidity (Wilker et al. 2012), according to the following formula:

1 $$ \begin{array}{l}\mathrm{Apparent}\kern0.5em \mathrm{temperature}=-2.653+\left[0.994\times 24\hbox{-} \mathrm{h}\kern0.5em \mathrm{mean}\kern0.5em \mathrm{air}\kern0.5em \mathrm{temperature}\left({}^{\circ}\mathrm{C}\right)\right]+\hfill \\ {}\left[0.0153\times 24\hbox{-} \mathrm{h}\kern0.5em \mathrm{mean}\kern0.5em \mathrm{dew}\kern0.5em \mathrm{point}\kern0.5em \mathrm{temperature}{\left({}^{\circ}\mathrm{C}\right)}^2\right]\hfill \end{array} $$

### Statistical analysis

We used ANOVA to compare the average 24-h PM_2.5_ concentrations across the five New York City boroughs over the study periods. Because there were no differences observed across boroughs, we calculated overall 24-h PM_2.5_ concentrations for New York City by averaging borough-specific 24-h PM_2.5_ concentrations. We calculated effects for single-day as well as multiple-day lags (lag 0 to lag 5 and lag 0–1 to lag 0–5) for 24-h PM_2.5_ concentrations for each city during the three 4-week periods.

Next, we compared daily mortality counts in different time periods using Poisson regression with adjustment for day of the week and daily average temperature. We first compared daily mortality counts in the 4-week period of July 2002 to those in the matched periods in 2001 and 2003. We then compared mortality counts on the wildfire-impacted days in 2002 (July 7–16) to those on the matched weekdays in 2001 and 2003. Finally, we compared the mortality rates on the non-impacted days in 2002 (July 1–6, 17–28) to those on the matched weekdays in 2001 and 2003. These analyses were done separately for Greater Boston and New York City.

For each city, we assessed the associations between daily mortality and PM_2.5_ concentrations in each of the three 4-week periods, and in all three 4-week periods combined, using generalized linear models. The endpoints considered included the daily counts for total mortality, cardiovascular mortality, and respiratory mortality. Often, counts data display greater variability than would be expected based on a Poisson distribution. This feature is referred to as overdispersion (Dean and Lawless 1989). We examined whether there was overdispersion in mortality data for the two cities during the study periods using the scaled deviances and found that there was no overdispersion in the data, except for the data from Greater Boston in July 2002. We thus fitted a negative binomial regression model to the data from Greater Boston in 2002 to account for overdispersion, and we fitted Poisson regression models to the rest of the data. We also performed Poisson regression modeling on the combined data across the three 4-week periods (2001 to 2003) for each city. Covariates considered in the single-period models were apparent temperature, week of the month, weekend, and holiday (Independence Day), as they were identified as potential covariates in the literature. We also included year as a covariate in the analyses on the combined data across the three periods. The general form of the models is shown as follows:

2 $$ \log \left\{E(Y)\right\}={\beta}_0+{\beta}_1{\mathrm{PM}}_{2.5}+{\beta}_2\mathrm{Appt}+{\beta}_3\mathrm{week}+{\beta}_4\mathrm{weekend}+{\beta}_5\mathrm{holiday}+{\beta}_6\mathrm{year} $$

*Y* denotes the daily mortality counts, PM_2.5_ represents the city-level 24-h PM_2.5_ concentration (μg/m^3^), Appt denotes the daily apparent temperature (°C), week is an indicator variable with four levels specifying the week of the month during the study periods, weekend/holiday is an indicator variable for weekend days or holidays, and year is an indicator variable with three levels denoting the years (2001 to 2003).

To combine the analyses for Greater Boston and New York City, we fit generalized linear mixed models considering city as the random effect. The endpoints and covariates were the same as in individual city analyses. There was no overdispersion in combined-city data as assessed by the scaled deviances; therefore, we fitted Poisson regression models to the data from single periods and data from combined periods. The mixed effect models have a general form as:

3 $$ \log \left\{E(Y)\right\}={\beta}_0+{\beta}_1{\mathrm{PM}}_{2.5}+{\beta}_2\mathrm{Appt}+{\beta}_3\mathrm{week}+{\beta}_4\mathrm{weekend}+{\beta}_5\mathrm{holiday}+{\beta}_6\mathrm{year}+{\gamma}_k $$

*γ*_*k*_ is a random intercept denoting the random effect of city *k* (*k* = 1, 2).

Using the models described previously, we individually tested the effect of PM_2.5_ on daily mortality at different lag times. We estimated relative risks (RRs) for a 10-μg/m^3^ increase in PM_2.5_ concentrations, 95 % confidence intervals (CIs), and the *P* values for all PM_2.5_ lags.

All analyses were performed using SAS version 9.3 (SAS Institute Inc., Cary, NC, USA) software packages.

## Results

### PM_2.5_ concentrations in Greater Boston and New York City

The 2002 Quebec wildfires significantly increased airborne PM_2.5_ in both Greater Boston and New York City over multiple days beginning on Saturday, July 6, and extending into Wednesday, July 10, with the largest PM_2.5_ impacts occurring on Sunday, July 7. As reflected in Fig. 1, similar hourly peak PM_2.5_ concentrations in excess of 130 μg/m^3^ were measured in both Boston and New York City, although the maximum hourly PM_2.5_ concentrations occurred about 12 h earlier in New York City than in the Boston area (8 to 11 a.m. on the morning of Sunday, July 7, versus 7 to 11 p.m. in the evening of Sunday, July 7). Maximum 24-h concentrations of 64.2 and 86.4 μg/m^3^ were recorded by daily monitors on July 7 in the Greater Boston area and the New York City boroughs, respectively. These maximum 24-h concentrations are approximately four- to fivefold higher than the corresponding 4-week average concentrations for the July 2001 and July 2003 data collected at the same monitoring locations. Between Sunday, July 7, and Tuesday, July 9, 24-h average concentrations in excess of 50 μg/m^3^ were frequently observed at most Greater Boston area and New York City monitoring locations. As reflected in Table 1, although limited in duration, the Quebec wildfires contributed to higher average PM_2.5_ concentrations over the 4-week period of interest in July 2002 as compared to the matched periods in 2001 and 2003.

**Table 1:** Summary of calculated average 24-h PM_2.5_ concentrations for the Greater Boston area and the five New York City Boroughs

Average 24-h PM_2.5_ concentration	Greater Boston area	New York City borough				
		Manhattan	Brooklyn	Queens	Bronx	Staten Island
July 1–28, 2002						
Mean (SD)	23.0 (14.0)	27.9 (17.9)	27.3 (18.1)	25.3 (18.0)	26.0 (17.4)	25.2 (18.3)
Range	4.1–64.5	7.0–80.8	5.6–82.1	5.6–79.9	6.2–80.2	4.8–84.2
Matched period in 2001						
Mean (SD)	13.4 (5.8)	16.1 (8.4)	15.4 (8.7)	14.8 (7.9)	15.2 (8.4)	14.5 (7.8)
Range	5.7–30.0	5.1–38.1	5.0–39.8	5.0–37.6	4.4–38.3	4.9–33.1
Matched period in 2003						
Mean (SD)	19.8 (9.9)	21.4 (7.9)	20.8 (7.5)	21.0 (7.1)	20.5 (7.7)	19.8 (7.0)
Range	7.8–47.8	10.2–38.2	9.4–38.4	8.5–37.1	8.7–37.9	10.0–35.3

### Daily mortality counts across time periods

In Greater Boston, total natural-cause mortality averaged 32.5 deaths per day (SD = 7.4) for the 4-week period in July 2002, which was not statistically different from average daily deaths during the matched periods in 2001 and 2003 (*P* = 0.88 and 0.34, respectively), as assessed by Poisson regression (Table 2). There were also no differences in daily total mortality rates between wildfire-impacted days in July 2002 and matched days in 2001 and 2003 (*P* = 0.41 and 0.82, respectively). Similarly, total mortality rates during non-impacted days in July 2002 were comparable to those in matched days in 2001 and 2003 (*P* = 0.32 and 0.22, respectively).

**Table 2:** Daily total mortality for Greater Boston and New York City

Time periods	Daily mortality mean (SD)	Comparison of mortality rates (*P* values^a^)	
		2002 days and matched 2001 days	2002 days and matched 2003 days
Greater Boston			
Total days: July 1–28, 2002	32.5 (7.4)	0.88	0.34
Matched days in 2001	31.1 (4.2)		
Matched days in 2003	31.1 (5.5)		
Wildfire-impacted days: July 7–16, 2002	30.4 (6.1)	0.41	0.82
Matched days in 2001	31.1 (5.2)		
Matched days in 2003	30.6 (8.0)		
Non-impacted days: July 1–6, 17–28, 2002	33.6 (8.0)	0.32	0.22
Matched days in 2001	31.2 (3.8)		
Matched days in 2003	31.4 (3.7)		
New York City			
Total days: July 1–28, 2002	141.6 (15.0)	0.72	0.88
Matched days in 2001	140.2 (12.9)		
Matched days in 2003	141.1 (13.9)		
Wildfire-impacted days: July 7–16, 2002	140.9 (8.4)	0.29	0.78
Matched days in 2001	145.8 (10.9)		
Matched days in 2003	137.9 (12.4)		
Non-impacted days: July 1–6, 17–28, 2002	142.0 (17.9)	0.1	0.85
Matched days in 2001	137.1 (13.1)		
Matched days in 2003	142.8 (14.8)		

^a^ *P* values are from Poisson regression, adjusting for day of the week and average temperature

In New York City, total natural-cause mortality averaged 141.6 deaths per day (SD = 15.0) for the 4-week period in July 2002. Similar to the results for Greater Boston, daily total mortality rates in NYC did not differ between time periods in July 2002 and matched periods in 2001 and 2003.

### PM_2.5_ levels and daily mortality

**Fig. 2:**
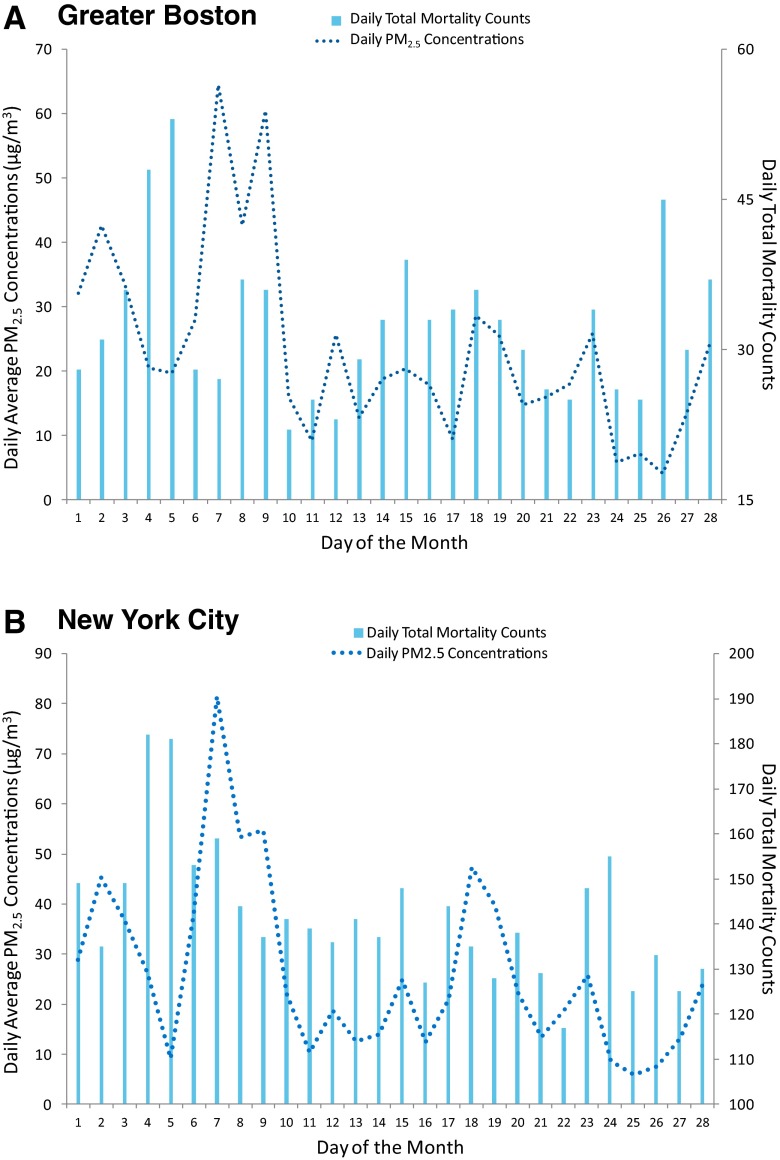
Time series of daily total mortality counts and PM_2.5_ concentrations in Greater Boston (**a**) and New York City (**b**) during the 4-week period in July 2002

Time series of daily total mortality counts and PM_2.5_ concentrations during the 4-week period in July 2002 are presented for Greater Boston (Fig. 2a) and New York City (Fig. 2b). Daily mortality rates peaked on July 4 and 5 in both cities, prior to any wildfire PM_2.5_ impact. These increases in mortality might have been attributable to the societal effect of the holiday (Independence Day) (Phillips et al. 2004). PM_2.5_ concentrations peaked on July 7, remained unusually high on July 8 and 9, and decreased to background levels afterwards. In Greater Boston, total mortality counts increased slightly on July 8 and 9, but were comparable to those in later days of the same month. In New York City, no increases in daily deaths were observed during or several days after the three high-PM_2.5_ days.

Using multivariate regression analyses, we evaluated the association between PM_2.5_ and daily mortality in each of the three 4-week periods as well as in all three periods combined (Online Resources Figure S1 and Tables S1–S3). We examined the effect of PM_2.5_ using 24-h average concentrations at lag 0 to lag 5 day, as well as using a 6-day moving average concentration. Single-city analyses yielded small effect estimates for PM_2.5_, which were close to the null value and bidirectional (>1 or <1). Most of the estimates did not achieve statistical significance; a few that did were likely due to multiple comparisons (dos Santos Silva I 1999, Chapter 13). Combined analyses showed similar results, i.e., there were no associations between PM_2.5_ and total mortality. We also examined cardiovascular and respiratory mortality and did not observe any effect of PM_2.5_ (Online Resources Tables S4-S9).

## Discussion

Our study evaluated whether daily mortality rates were increased by substantially elevated PM_2.5_ in Greater Boston and New York City during a regional air pollution episode in early July 2002, in which smoke plumes from Canadian wild forest fires blanketed the US eastern seaboard. We found that during the event and several days following it, daily mortality rates were unaffected by marked increases in PM_2.5_ concentrations. We also evaluated matching July days in the years prior to and after 2002 and did not observe differences in daily mortality over those 3 years. The data did not show an association between short-term exposures to increased ambient PM_2.5_ levels from wildfire smoke and increases in daily mortality.

The reported associations between short-term PM_2.5_ and daily mortality are subject to several uncertainties in interpretation. First, the reported effects of PM_2.5_ on daily mortality have varied widely across different areas; in some cities, increases in PM_2.5_ were in fact associated with statistically significant decreases in mortality (Franklin et al. 2007; Zanobetti and Schwartz 2009). Second, associations between PM_2.5_ and mortality were positive in some cities having lower levels of PM_2.5_, while risk estimates in the same study were negative for some cities with higher concentrations of PM_2.5_ (Franklin et al. 2007). These observations suggest that the observed associations between PM_2.5_ and mortality may be influenced by other factors, such as exposure measurement error and spatial heterogeneity (Sheppard et al. 2012). Third, none of the major components of PM_2.5_, including sulfate, carbon, and nitrate, show toxicity in laboratory exposures at low concentrations (Schlesinger 2007; Aben et al. 2002; Schlesinger and Cassee 2002; Valberg 2004). Fourth, PM_2.5_ is often highly correlated with gaseous co-pollutants, and associations between gaseous pollutants and mortality have also been observed in epidemiology studies (Stieb et al. 2002). Therefore, the confounding of ambient gaseous co-pollutants (including multiple hazardous air pollutants [HAPs]) on the observed association between PM_2.5_ and mortality cannot be ruled out. Fifth, mortality findings as to PM_2.5_ associations are often inconsistent with associations between PM_2.5_ and hospital admissions for specific causes. For example, a recent large-scale study in England and Wales reported a positive association between PM_2.5_ and mortality by various cardiovascular causes (Milojevic et al. 2014). However, in the same population, the study also reported generally negative associations between PM_2.5_ and emergency hospital admissions for cardiovascular causes, some of which reached statistical significance. Lastly, both anthropogenic PM_2.5_ emissions and mortality risk correlate with varying degrees of societal activity (Phillips et al. 1999; Muller-Nordhorn and Willich 2000; Peters et al. 2004; Tapia Granados 2005; Gronlund et al. 2014).

Thus, one interpretation of our null results might be that ambient PM_2.5_ is not associated with daily mortality in situations where the pollution increment is not confounded by possible variations in “societal stress” indices (e.g., daily noise levels, traffic counts, cell phone usage, electric power consumption, economic fluctuations, etc.) that could have effects on ambient PM_2.5_.

This study has the advantage of evaluating a natural experiment where the actual source of the PM_2.5_ was far away (~800 to 1000 miles). Annual average ambient concentrations of PM_2.5_ usually do not exceed 20 μg/m^3^ in the majority of US counties (US EPA 2009). Time-series studies rely on generally low concentrations of PM_2.5_ and fluctuations within a narrow range. In contrast, during the few days in July 2002, the general population in Greater Boston and New York City experienced two- to threefold increases in 24-h ambient PM_2.5_ concentrations. This wider range of exposure distribution increased the study power to detect a small effect, if any was present. Furthermore, as the smoke plume was generated by wildfires far distant from the study areas, the elevation in PM_2.5_ was not associated with local human activities or societal stress, minimizing the confounding effect by these factors. As compared to wildfires that occur much closer to populated areas, reactive gases created by the combustion process were likely attenuated, leaving primarily a PM_2.5_ exposure.

Our findings are consistent with other studies that evaluated the impact of elevated PM_2.5_ from wildfires on mortality. Vedal and Dutton (2006) examined daily mortality in Denver during 2 days in June 2002, in which hourly PM_2.5_ concentrations reached 200 μg/m^3^ due to wildfire smoke. No perceptible increases in mortality accompanied the abrupt and dramatic increases in PM_2.5_ concentrations. Emmanuel (2000) evaluated the health impact of a prolonged haze event in Singapore due to uncontrolled forest fires in Indonesia from the end of August to the first week of November 1997. No significant increases in mortality or in hospital admissions were observed during this period. In addition, during the same period, an increase in accidents and emergency attendance for haze-related conditions was observed. Similarly, Viswanathan et al. (2006) reported increases in hospital admissions for asthma, smoke inhalation, and eye irritation in San Diego in October 2003, when wildfires in Southern California caused dramatic increases in air pollution levels, including PM_2.5_; but, no significant increases in hospital admissions for chest pain/cardiac arrest were seen during the surveillance period. A recent study (Le et al. 2014) investigated hospital admissions for cardiovascular and respiratory causes among Medicare enrollees in the northeastern and mid-Atlantic regions of the USA affected by the smoke plume from the 2002 Quebec wildfires. Significant increases in hospital admissions for cardiovascular and respiratory causes were observed when the smoke plume was present compared to before the smoke plume arrived. However, these observed increases were independent of changes in ambient PM_2.5_ concentrations.

A recent systematic review on the physical health impact from non-occupational exposure to wildfire smoke evaluated 61 epidemiology studies of wildfire and human health in communities. The majority of studies focused on areas close to fire events and compared the risk of health outcomes either between periods with no fire events and periods during or after fire events, or between regions affected by wildfire smoke and unaffected regions (Liu et al. 2015). Twelve studies evaluated total mortality. While the majority reported a positive association with fire events, only three studies specifically assessed non-accidental mortality. Of these three studies, two that were conducted outside of the USA reported positive associations between wildfires and total non-accidental mortality, but did not measure PM_2.5_ during the fire events. The third study (Vedal and Dutton 2006), as discussed previously, reported null associations.

It is well recognized that wood smoke particles can differ in their chemical composition and physicochemical properties from other combustion-related PM types, such as traffic-related PM and coal combustion PM (Lighty et al. 2000; Naeher et al. 2007; Morandi and Ward 2010; Kocbach Bolling et al. 2009). While a topic of intensive research in recent years, the relative toxicity of source-specific PM, including wood smoke PM, remains highly uncertain. Some studies find that wood smoke particles from residential wood burning and wildland fires may be less toxic than other types of ambient PM emissions. For example, two epidemiological studies that used data from PM_2.5_ source apportionments, namely the Mar et al. (2006) analysis of mortality in Phoenix, AZ, and the Ito et al. (2006) analysis of mortality in Washington, DC, failed to find consistent, statistically significant associations for wood smoke or biomass/wood combustion factors. Instead, these studies reported evidence of larger, more consistent associations for other PM source types, including coal combustion primary PM_2.5_, secondary sulfates, and traffic-related PM_2.5_. Interestingly, as summarized by Stanek et al. (2011), the Ito et al. (2006) study reported more consistent evidence of decreased mortality for their wood smoke factor.

Other studies have found that wood smoke particles from forest fires and prescribed fires may be of similar toxicity, if not of greater toxicity, than those of other ambient PM source types. For example, Sarnat et al. (2008) conducted an epidemiological study of the region in Atlanta, GA, where prescribed forest management burning in the summer and residential wood burning in the winter result in a sizeable year-round wood smoke PM_2.5_ contribution. They conducted analyses using several different kinds of source apportionment estimates and observed similarities in significance and lag structure between responses for a biomass burning or wood smoke source factor and cardiovascular disease (CVD)-related emergency department (ED) visits, as for a diesel source factor and CVD-related ED visits. In addition, while the human health relevance of in vitro and mouse bioassays is uncertain, both Myatt et al. (2011) and Wegesser et al. (2009) reported greater biological activity of wood smoke particles as compared to more typical ambient PM_2.5_ samples collected in the same areas under normal (non-fire) conditions.

As these examples show, the evidence regarding the relative potency of wood smoke PM versus other ambient PM is inconsistent (Naeher et al. 2007; Morandi and Ward 2010). Moreover, it is possible that there may be variability in the relative potency of different types of wood smoke PM due to differences in its composition and other properties (e.g., particle size) depending on vegetation type and combustion conditions (Liu et al. 2015).

Our study has several limitations. First, given the natural experiment nature, our study period is short and thus may have limited power to detect a small effect. However, we do not believe the null findings can be attributed solely to limited power. This is because the changes in PM_2.5_ levels during this short period of time were far larger than those of the other time-series studies conducted in the USA. Also, we looked at daily mortality in two highly populated cities. Based on multicity analyses in the USA, a 10-μg/m^3^ increase in 24-h PM_2.5_ concentration at lag 0–1 days is associated with 0.98–2.76 % increases in total mortality (Zanobetti and Schwartz 2008 and 2009). Given the 40–50-μg/m^3^ increases in 24-h PM_2.5_ concentrations in these two cities, approximately 4–14 % increases in total mortality would have been expected. As discussed previously, other studies of wildfire PM_2.5_ have reported null findings on mortality (Vedal and Dutton 2006; Emmanuel 2000), but significant effects on hospitalization and emergency department visits for respiratory causes (Viswanathan et al. 2006; Emmanuel 2000). In the literature, the range of excess risk estimates for respiratory hospitalization associated with elevated PM_2.5_ are comparable to that of total mortality (US EPA 2009). Thus, it is not likely that the null results associated with elevated PM_2.5_ from wildfires are a result of a short study duration for either the present or prior studies. Second, we relied on several centrally located air monitors for PM_2.5_ measurement data and used spatially averaged overall values to represent daily PM_2.5_ concentrations in two large geographical areas (Greater Boston and New York City). However, measurement data from monitors within the same area suggest there is limited spatial variation in PM_2.5_ within the area. Third, during the few days in which PM_2.5_ concentrations increased dramatically, there was a visible haze in the study areas. People, especially the elderly and those with respiratory conditions, were more likely to stay indoors. Indoor ambient PM_2.5_ concentrations, particularly in buildings with central air conditioning, could be substantially lower than outdoor concentrations. This may contribute, at least partially, to the null results observed in this study. Lastly, our study is ecological in nature; thus, our ability to make causal inferences at the individual level is limited. However, our findings add to the growing literature on the health impact of PM_2.5_ from wildfires and, more generally, on the observed association between short-term exposures to ambient PM_2.5_ from other combustion sources and daily mortality.

Recent editorials have stressed the uncertainties regarding whether statistical associations between PM_2.5_ and mortality are causal (Dominici et al. 2014), and the scientific merit of null results (Wilcox 2014). As epidemiological research on air pollution plays a critical role in policy making (McClellan 2012), our findings should be considered in assessments conducted for regulation such as the short-term PM_2.5_ National Ambient Air Quality Standards (NAAQS).

## Conclusions

We found that substantial short-term increases in PM_2.5_ concentrations from forest fire smoke were not associated with increases in daily mortality in Greater Boston or New York City. While focused on the impacts of PM_2.5_ from wildfires, our results bear on the interpretation of associations between short-term PM_2.5_ exposures and daily mortality.

## Electronic supplementary material

Below is the link to the electronic supplementary material.

ESM 1 (DOCX 109 kb)
