# Supplementary material for: Long-range fine particulate matter from the 2002 Quebec forest fires and daily mortality in Greater Boston and New York City
Source: Air Qual Atmos Health. 2015 Feb 28;9(3):213–21. doi: 10.1007/s11869-015-0332-9 (PMC4837205; doi:10.1007/s11869-015-0332-9)
Supplement: Supplementary file 1 — (DOCX 109 kb) [file 11869_2015_332_MOESM1_ESM.docx]

**Long-Range Fine Particulate Matter from the 2002 Quebec Forest Fires and Daily Mortality in Greater Boston and New York City**

Ke Zu*, Ge Tao, Christopher Long, Julie Goodman, and Peter Valberg

*Gradient, 20 University Road Cambridge, MA 02138.*

*Corresponding Author; email: kzu@gradientcorp.com

**Figure. S1.** Relative risks of mortality associated with a 10 μg/m^3^ increase in PM_2.5_ concentrations in Greater Boston, New York City, and the two areas combined.

Relative risks were estimated from Poisson regression or negative binomial regression (when there was dispersion), adjusted for holiday, weekend, week of the month, and apparent temperature, in single-year analyses, and adjusted for year, in addition to those covariates, in the combined analysis. Random-effects models were used to combine Greater Boston and New York City.

**Table S1. Multivariate Regression Analyses^a^ of Daily Total Mortality and PM_2.5_ in Greater Boston between 2001-2003**

| **PM_2.5_ Lags** | **2001** | | | | **2002** | | | | **2003** | | | | **2001-2003 Combined** | | | |
| --- | --- | --- | --- | --- | --- | --- | --- | --- | --- | --- | --- | --- | --- | --- | --- | --- |
|  | **RR^b^** | **95% CI** | | **P** | **RR** | **95% CI** | | **P** | **RR** | **95% CI** | | **P** | **RR** | **95% CI** | | **P** |
| Lag 0 | 1.06 | 0.87 | 1.29 | 0.58 | 0.99 | 0.92 | 1.06 | 0.78 | 0.99 | 0.86 | 1.15 | 0.91 | 0.99 | 0.94 | 1.04 | 0.62 |
| Lag 1 | 1.03 | 0.90 | 1.18 | 0.66 | 0.99 | 0.93 | 1.06 | 0.81 | 0.92 | 0.79 | 1.07 | 0.26 | 0.98 | 0.94 | 1.03 | 0.50 |
| Lag 2 | 0.95 | 0.84 | 1.06 | 0.37 | 1.00 | 0.93 | 1.06 | 0.93 | 0.95 | 0.83 | 1.08 | 0.42 | 0.99 | 0.94 | 1.03 | 0.52 |
| Lag 3 | 0.86 | 0.78 | 0.95 | 0.00 | 0.97 | 0.90 | 1.04 | 0.39 | 1.02 | 0.92 | 1.14 | 0.66 | 0.98 | 0.94 | 1.02 | 0.39 |
| Lag 4 | 1.10 | 0.96 | 1.27 | 0.17 | 0.96 | 0.89 | 1.02 | 0.19 | 0.99 | 0.91 | 1.08 | 0.87 | 0.98 | 0.94 | 1.02 | 0.27 |
| Lag 5 | 1.11 | 1.00 | 1.24 | 0.05 | 0.96 | 0.89 | 1.04 | 0.33 | 0.91 | 0.84 | 1.00 | 0.05 | 0.97 | 0.92 | 1.01 | 0.12 |
| Lag 0-1 | 1.07 | 0.87 | 1.32 | 0.52 | 0.99 | 0.91 | 1.07 | 0.75 | 0.86 | 0.66 | 1.12 | 0.27 | 0.98 | 0.92 | 1.04 | 0.45 |
| Lag 0-2 | 0.99 | 0.81 | 1.21 | 0.92 | 0.99 | 0.90 | 1.09 | 0.77 | 0.79 | 0.58 | 1.06 | 0.12 | 0.97 | 0.91 | 1.04 | 0.37 |
| Lag 0-3 | 0.86 | 0.70 | 1.07 | 0.19 | 0.96 | 0.85 | 1.08 | 0.54 | 0.87 | 0.62 | 1.20 | 0.39 | 0.96 | 0.89 | 1.03 | 0.27 |
| Lag 0-4 | 0.90 | 0.68 | 1.19 | 0.45 | 0.91 | 0.78 | 1.06 | 0.22 | 0.92 | 0.70 | 1.19 | 0.52 | 0.94 | 0.87 | 1.02 | 0.16 |
| Lag 0-5 | 1.02 | 0.74 | 1.42 | 0.90 | 0.81 | 0.66 | 1.00 | 0.05 | 0.84 | 0.65 | 1.08 | 0.18 | 0.92 | 0.84 | 1.01 | 0.06 |

Notes:

(a) Results for 2002 were from negative binomial regression models due to overdispersion; others were from Poisson regression models. Covariates adjusted in the Poisson regression models included apparent temperature, week, weekend, and holiday, in single-year analyses, and year, in multi-year analyses.

(b) RR was calculated for a 10 µg/m3 increase in PM_2.5_ concentrations.

**Table S2. Multivariate Regression Analyses^a^ of Daily Total Mortality and PM_2.5_ in New York City between 2001-2003**

| **PM_2.5_ Lags** | **2001** | | | | **2002** | | | | **2003** | | | | **2001-2003 Combined** | | | |
| --- | --- | --- | --- | --- | --- | --- | --- | --- | --- | --- | --- | --- | --- | --- | --- | --- |
|  | **RR^b^** | **95% CI** | | **P** | **RR** | **95% CI** | | **P** | **RR** | **95% CI** | | **P** | **RR** | **95% CI** | | **P** |
| Lag 0 | 1.04 | 0.95 | 1.13 | 0.41 | 1.00 | 0.98 | 1.02 | 0.94 | 0.98 | 0.90 | 1.06 | 0.57 | 1.00 | 0.98 | 1.02 | 0.82 |
| Lag 1 | 1.01 | 0.96 | 1.06 | 0.76 | 1.01 | 0.99 | 1.03 | 0.41 | 1.00 | 0.93 | 1.06 | 0.87 | 1.00 | 0.98 | 1.02 | 0.76 |
| Lag 2 | 0.97 | 0.87 | 1.09 | 0.22 | 1.00 | 0.98 | 1.02 | 0.77 | 0.96 | 0.92 | 1.01 | 0.11 | 0.99 | 0.97 | 1.01 | 0.30 |
| Lag 3 | 1.01 | 0.97 | 1.05 | 0.59 | 1.00 | 0.98 | 1.02 | 0.98 | 1.00 | 0.96 | 1.04 | 0.84 | 1.01 | 0.99 | 1.02 | 0.55 |
| Lag 4 | 1.00 | 0.95 | 1.04 | 0.92 | 1.00 | 0.97 | 1.02 | 0.81 | 1.02 | 0.98 | 1.05 | 0.33 | 1.00 | 0.99 | 1.02 | 0.66 |
| Lag 5 | 1.01 | 0.98 | 1.05 | 0.59 | 1.01 | 0.99 | 1.03 | 0.32 | 1.00 | 0.97 | 1.04 | 0.90 | 1.00 | 0.99 | 1.02 | 0.61 |
| Lag 0-1 | 1.03 | 0.95 | 1.12 | 0.50 | 1.01 | 0.98 | 1.03 | 0.66 | 0.95 | 0.82 | 1.10 | 0.49 | 1.00 | 0.98 | 1.03 | 0.94 |
| Lag 0-2 | 0.99 | 0.91 | 1.08 | 0.79 | 1.01 | 0.98 | 1.04 | 0.63 | 0.89 | 0.78 | 1.00 | 0.06 | 0.99 | 0.97 | 1.02 | 0.63 |
| Lag 0-3 | 1.01 | 0.91 | 1.11 | 0.90 | 1.01 | 0.97 | 1.04 | 0.70 | 0.91 | 0.81 | 1.03 | 0.15 | 1.00 | 0.97 | 1.03 | 0.89 |
| Lag 0-4 | 1.00 | 0.91 | 1.11 | 0.95 | 1.01 | 0.97 | 1.05 | 0.78 | 0.99 | 0.89 | 1.09 | 0.77 | 1.00 | 0.97 | 1.03 | 0.96 |
| Lag 0-5 | 1.02 | 0.91 | 1.13 | 0.75 | 1.02 | 0.97 | 1.07 | 0.51 | 0.99 | 0.89 | 1.10 | 0.85 | 1.00 | 0.97 | 1.04 | 0.82 |

Notes:

(a) Covariates adjusted in the Poisson regression models included apparent temperature, week, weekend, and holiday, in single-year analyses, and year, in multi-year analyses.

(b) RR was calculated for a 10 µg/m3 increase in PM_2.5_ concentrations.

**Table S3. Multivariate Regression Analyses^a^ of Daily Total Mortality and PM_2.5_ in Greater Boston and New York City between 2001-2003**

| **PM_2.5_ Lags** | **2001** | | | | **2002** | | | | **2003** | | | | **2001-2003 Combined** | | | |
| --- | --- | --- | --- | --- | --- | --- | --- | --- | --- | --- | --- | --- | --- | --- | --- | --- |
|  | **RR^b^** | **95% CI** | | **P** | **RR** | **95% CI** | | **P** | **RR** | **95% CI** | | **P** | **RR** | **95% CI** | | **P** |
| Lag 0 | 1.04 | 0.98 | 1.12 | 0.22 | 1.00 | 0.98 | 1.02 | 0.77 | 0.98 | 0.92 | 1.05 | 0.55 | 1.00 | 0.98 | 1.01 | 0.61 |
| Lag 1 | 1.01 | 0.96 | 1.05 | 0.70 | 1.00 | 0.98 | 1.03 | 0.66 | 0.98 | 0.93 | 1.04 | 0.52 | 1.00 | 0.98 | 1.02 | 0.92 |
| Lag 2 | 0.97 | 0.93 | 1.01 | 0.11 | 1.00 | 0.98 | 1.02 | 0.89 | 0.96 | 0.92 | 1.00 | 0.08 | 0.99 | 0.98 | 1.01 | 0.22 |
| Lag 3 | 1.00 | 0.97 | 1.04 | 0.94 | 1.00 | 0.98 | 1.02 | 0.81 | 1.00 | 0.96 | 1.04 | 0.98 | 1.00 | 0.99 | 1.02 | 0.65 |
| Lag 4 | 1.00 | 0.96 | 1.04 | 0.95 | 0.99 | 0.97 | 1.02 | 0.55 | 1.01 | 0.98 | 1.04 | 0.40 | 1.00 | 0.99 | 1.02 | 0.91 |
| Lag 5 | 1.02 | 0.98 | 1.05 | 0.34 | 1.01 | 0.98 | 1.03 | 0.57 | 0.99 | 0.96 | 1.02 | 0.56 | 1.00 | 0.99 | 1.02 | 0.94 |
| Lag 0-1 | 1.04 | 0.96 | 1.12 | 0.33 | 1.00 | 0.98 | 1.03 | 0.93 | 0.93 | 0.82 | 1.05 | 0.22 | 1.00 | 0.98 | 1.02 | 0.81 |
| Lag 0-2 | 0.99 | 0.92 | 1.06 | 0.72 | 1.00 | 0.97 | 1.03 | 0.89 | 0.87 | 0.77 | 0.98 | 0.02 | 0.99 | 0.97 | 1.01 | 0.42 |
| Lag 0-3 | 0.99 | 0.91 | 1.08 | 0.80 | 1.00 | 0.97 | 1.03 | 0.99 | 0.91 | 0.81 | 1.02 | 0.10 | 0.99 | 0.97 | 1.02 | 0.64 |
| Lag 0-4 | 0.99 | 0.91 | 1.08 | 0.81 | 1.00 | 0.96 | 1.04 | 0.84 | 0.97 | 0.89 | 1.07 | 0.57 | 0.99 | 0.97 | 1.02 | 0.71 |
| Lag 0-5 | 1.01 | 0.92 | 1.11 | 0.80 | 1.00 | 0.95 | 1.05 | 1.00 | 0.97 | 0.88 | 1.06 | 0.46 | 0.99 | 0.96 | 1.03 | 0.75 |

Notes:

(a) Random-effects models were used to combine the Poisson regression analyses between Greater Boston and New York City. Covariates adjusted in the Poisson regression models included apparent temperature, week, weekend, and holiday, in single-year analyses, and year, in multi-year analyses.

(b) RR was calculated for a 10 µg/m3 increase in PM_2.5_ concentrations.

**Table S4. Multivariate Regression Analyses^a^ of Daily Cardiovascular Mortality and PM_2.5_ in Greater Boston between 2001-2003**

| **PM_2.5_ Lags** | **2001** | | | | **2002** | | | | **2003** | | | | **2001-2003 Combined** | | | |
| --- | --- | --- | --- | --- | --- | --- | --- | --- | --- | --- | --- | --- | --- | --- | --- | --- |
|  | **RR^b^** | **95% CI** | | **P** | **RR** | **95% CI** | | **P** | **RR** | **95% CI** | | **P** | **RR** | **95% CI** | | **P** |
| Lag 0 | 0.76 | 0.46 | 1.24 | 0.27 | 0.97 | 0.87 | 1.09 | 0.62 | 0.87 | 0.69 | 1.11 | 0.27 | 0.97 | 0.87 | 1.07 | 0.51 |
| Lag 1 | 0.87 | 0.60 | 1.24 | 0.44 | 1.06 | 0.96 | 1.17 | 0.26 | 0.93 | 0.71 | 1.21 | 0.59 | 1.04 | 0.95 | 1.14 | 0.38 |
| Lag 2 | 0.82 | 0.60 | 1.11 | 0.19 | 1.01 | 0.91 | 1.12 | 0.85 | 0.92 | 0.74 | 1.15 | 0.48 | 1.01 | 0.92 | 1.10 | 0.86 |
| Lag 3 | 0.82 | 0.60 | 1.12 | 0.20 | 1.03 | 0.91 | 1.16 | 0.64 | 0.98 | 0.82 | 1.19 | 0.87 | 1.05 | 0.97 | 1.14 | 0.23 |
| Lag 4 | 1.54 | 1.10 | 2.14 | 0.01 | 0.91 | 0.80 | 1.03 | 0.15 | 0.90 | 0.78 | 1.04 | 0.15 | 0.99 | 0.91 | 1.08 | 0.84 |
| Lag 5 | 1.24 | 0.92 | 1.67 | 0.15 | 0.90 | 0.79 | 1.03 | 0.12 | 0.81 | 0.71 | 0.94 | 0.00 | 0.93 | 0.85 | 1.02 | 0.15 |
| Lag 0-1 | 0.72 | 0.42 | 1.22 | 0.22 | 1.03 | 0.90 | 1.18 | 0.66 | 0.70 | 0.44 | 1.10 | 0.12 | 1.01 | 0.89 | 1.14 | 0.86 |
| Lag 0-2 | 0.67 | 0.40 | 1.11 | 0.12 | 1.04 | 0.88 | 1.21 | 0.66 | 0.60 | 0.36 | 1.01 | 0.06 | 1.02 | 0.89 | 1.16 | 0.83 |
| Lag 0-3 | 0.58 | 0.34 | 1.00 | 0.05 | 1.06 | 0.87 | 1.30 | 0.54 | 0.63 | 0.35 | 1.11 | 0.11 | 1.05 | 0.91 | 1.22 | 0.48 |
| Lag 0-4 | 0.70 | 0.35 | 1.42 | 0.33 | 1.00 | 0.77 | 1.30 | 1.00 | 0.65 | 0.42 | 1.01 | 0.05 | 1.05 | 0.89 | 1.23 | 0.59 |
| Lag 0-5 | 0.87 | 0.37 | 2.07 | 0.75 | 0.85 | 0.58 | 1.25 | 0.42 | 0.55 | 0.37 | 0.81 | 0.00 | 1.00 | 0.83 | 1.20 | 0.99 |

Notes:

(a) Results for 2002 were from negative binomial regression models due to overdispersion; others were from Poisson regression models. Covariates adjusted in the Poisson regression models included apparent temperature, week, weekend, and holiday, in single-year analyses, and year, in multi-year analyses.

(b) RR was calculated for a 10 µg/m3 increase in PM_2.5_ concentrations.

**Table S5. Multivariate Regression Analyses^a^ of Daily Cardiovascular Mortality and PM_2.5_ in New York City between 2001-2003**

| **PM_2.5_ Lags** | **2001** | | | | **2002** | | | | **2003** | | | | **2001-2003 Combined** | | | |
| --- | --- | --- | --- | --- | --- | --- | --- | --- | --- | --- | --- | --- | --- | --- | --- | --- |
|  | **RR^b^** | **95% CI** | | **P** | **RR** | **95% CI** | | **P** | **RR** | **95% CI** | | **P** | **RR** | **95% CI** | | **P** |
| Lag 0 | 1.09 | 0.96 | 1.25 | 0.19 | 1.01 | 0.98 | 1.05 | 0.54 | 0.97 | 0.86 | 1.09 | 0.60 | 1.01 | 0.97 | 1.04 | 0.74 |
| Lag 1 | 1.05 | 0.97 | 1.14 | 0.22 | 1.01 | 0.98 | 1.05 | 0.54 | 1.07 | 0.98 | 1.16 | 0.12 | 1.02 | 0.99 | 1.05 | 0.16 |
| Lag 2 | 0.97 | 0.90 | 1.05 | 0.50 | 1.00 | 0.97 | 1.04 | 0.81 | 0.94 | 0.88 | 1.01 | 0.09 | 1.00 | 0.97 | 1.02 | 0.72 |
| Lag 3 | 0.99 | 0.93 | 1.05 | 0.72 | 1.01 | 0.98 | 1.05 | 0.54 | 1.00 | 0.95 | 1.06 | 1.00 | 1.01 | 0.98 | 1.03 | 0.54 |
| Lag 4 | 0.98 | 0.91 | 1.06 | 0.63 | 1.01 | 0.98 | 1.05 | 0.49 | 1.04 | 1.00 | 1.08 | 0.06 | 1.01 | 0.99 | 1.04 | 0.35 |
| Lag 5 | 1.01 | 0.96 | 1.07 | 0.67 | 1.02 | 0.98 | 1.06 | 0.29 | 1.02 | 0.97 | 1.07 | 0.53 | 1.01 | 0.98 | 1.04 | 0.49 |
| Lag 0-1 | 1.12 | 0.99 | 1.28 | 0.08 | 1.02 | 0.97 | 1.06 | 0.47 | 1.15 | 0.94 | 1.40 | 0.19 | 1.02 | 0.98 | 1.06 | 0.26 |
| Lag 0-2 | 1.05 | 0.92 | 1.20 | 0.48 | 1.02 | 0.97 | 1.07 | 0.50 | 0.94 | 0.78 | 1.13 | 0.50 | 1.01 | 0.97 | 1.06 | 0.54 |
| Lag 0-3 | 1.03 | 0.88 | 1.21 | 0.71 | 1.02 | 0.97 | 1.08 | 0.41 | 0.96 | 0.80 | 1.14 | 0.64 | 1.02 | 0.97 | 1.06 | 0.44 |
| Lag 0-4 | 1.01 | 0.85 | 1.19 | 0.93 | 1.03 | 0.97 | 1.10 | 0.31 | 1.06 | 0.92 | 1.22 | 0.41 | 1.03 | 0.98 | 1.07 | 0.30 |
| Lag 0-5 | 1.03 | 0.86 | 1.22 | 0.78 | 1.06 | 0.98 | 1.15 | 0.13 | 1.07 | 0.93 | 1.23 | 0.33 | 1.03 | 0.98 | 1.09 | 0.22 |

Notes:

(a) Covariates adjusted in the Poisson regression models included apparent temperature, week, weekend, and holiday, in single-year analyses, and year, in multi-year analyses.

(b) RR was calculated for a 10 µg/m3 increase in PM_2.5_ concentrations.

**Table S6. Multivariate Regression Analyses^a^ of Daily Cardiovascular Mortality and PM_2.5_ in Greater Boston and New York City between 2001-2003**

| **PM_2.5_ Lags** | **2001** | | | | **2002** | | | | **2003** | | | | **2001-2003 Combined** | | | |
| --- | --- | --- | --- | --- | --- | --- | --- | --- | --- | --- | --- | --- | --- | --- | --- | --- |
|  | **RR^b^** | **95% CI** | | **P** | **RR** | **95% CI** | | **P** | **RR** | **95% CI** | | **P** | **RR** | **95% CI** | | **P** |
| Lag 0 | 1.07 | 0.96 | 1.18 | 0.22 | 1.01 | 0.98 | 1.04 | 0.70 | 0.92 | 0.83 | 1.02 | 0.11 | 1.00 | 0.97 | 1.03 | 0.91 |
| Lag 1 | 1.04 | 0.98 | 1.11 | 0.23 | 1.01 | 0.98 | 1.05 | 0.41 | 1.05 | 0.97 | 1.14 | 0.26 | 1.02 | 1.00 | 1.05 | 0.08 |
| Lag 2 | 0.97 | 0.91 | 1.03 | 0.28 | 1.00 | 0.97 | 1.04 | 0.81 | 0.94 | 0.88 | 1.01 | 0.08 | 1.00 | 0.98 | 1.02 | 0.76 |
| Lag 3 | 0.99 | 0.94 | 1.03 | 0.55 | 1.01 | 0.98 | 1.04 | 0.36 | 1.00 | 0.95 | 1.05 | 0.92 | 1.01 | 0.99 | 1.03 | 0.23 |
| Lag 4 | 1.00 | 0.94 | 1.06 | 0.90 | 1.01 | 0.98 | 1.04 | 0.62 | 1.03 | 0.98 | 1.07 | 0.25 | 1.01 | 0.99 | 1.03 | 0.31 |
| Lag 5 | 1.02 | 0.98 | 1.07 | 0.34 | 1.01 | 0.98 | 1.05 | 0.40 | 0.99 | 0.95 | 1.04 | 0.81 | 1.00 | 0.98 | 1.03 | 0.74 |
| Lag 0-1 | 1.10 | 0.98 | 1.22 | 0.10 | 1.01 | 0.98 | 1.05 | 0.47 | 0.98 | 0.81 | 1.18 | 0.83 | 1.02 | 0.99 | 1.05 | 0.28 |
| Lag 0-2 | 1.02 | 0.92 | 1.14 | 0.66 | 1.02 | 0.97 | 1.06 | 0.50 | 0.86 | 0.72 | 1.02 | 0.10 | 1.01 | 0.98 | 1.05 | 0.54 |
| Lag 0-3 | 1.00 | 0.88 | 1.13 | 0.99 | 1.02 | 0.98 | 1.08 | 0.34 | 0.90 | 0.76 | 1.06 | 0.21 | 1.02 | 0.98 | 1.06 | 0.30 |
| Lag 0-4 | 1.00 | 0.87 | 1.13 | 0.95 | 1.03 | 0.97 | 1.09 | 0.29 | 0.99 | 0.87 | 1.14 | 0.92 | 1.03 | 0.99 | 1.07 | 0.19 |
| Lag 0-5 | 1.03 | 0.90 | 1.18 | 0.68 | 1.06 | 0.98 | 1.14 | 0.15 | 0.99 | 0.86 | 1.13 | 0.85 | 1.03 | 0.99 | 1.08 | 0.18 |

Notes:

(a) Random-effects models were used to combine the Poisson regression analyses between Greater Boston and New York City. Covariates adjusted in the Poisson regression models included apparent temperature, week, weekend, and holiday, in single-year analyses, and year, in multi-year analyses.

(b) RR was calculated for a 10 µg/m3 increase in PM_2.5_ concentrations.

**Table S7. Multivariate Regression Analyses^a^ of Daily Respiratory Mortality and PM_2.5_ in Greater Boston between 2001-2003**

| **PM_2.5_ Lags** | **2001** | | | | **2002** | | | | **2003** | | | | **2001-2003 Combined** | | | |
| --- | --- | --- | --- | --- | --- | --- | --- | --- | --- | --- | --- | --- | --- | --- | --- | --- |
|  | **RR^b^** | **95% CI** | | **P** | **RR** | **95% CI** | | **P** | **RR** | **95% CI** | | **P** | **RR** | **95% CI** | | **P** |
| Lag 0 | 1.24 | 0.56 | 2.76 | 0.60 | 0.97 | 0.78 | 1.22 | 0.82 | 0.98 | 0.72 | 1.33 | 0.89 | 0.97 | 0.82 | 1.15 | 0.73 |
| Lag 1 | 1.36 | 0.82 | 2.28 | 0.23 | 0.98 | 0.79 | 1.21 | 0.82 | 0.63 | 0.47 | 0.86 | 0.00 | 0.95 | 0.81 | 1.12 | 0.57 |
| Lag 2 | 0.93 | 0.57 | 1.51 | 0.77 | 0.94 | 0.75 | 1.18 | 0.60 | 0.98 | 0.73 | 1.31 | 0.87 | 0.96 | 0.83 | 1.12 | 0.63 |
| Lag 3 | 0.74 | 0.46 | 1.19 | 0.21 | 0.87 | 0.66 | 1.14 | 0.31 | 1.12 | 0.88 | 1.42 | 0.36 | 0.93 | 0.80 | 1.08 | 0.37 |
| Lag 4 | 0.48 | 0.26 | 0.89 | 0.02 | 1.02 | 0.81 | 1.29 | 0.87 | 0.99 | 0.81 | 1.21 | 0.91 | 0.91 | 0.79 | 1.06 | 0.24 |
| Lag 5 | 1.05 | 0.67 | 1.64 | 0.83 | 0.94 | 0.73 | 1.23 | 0.67 | 0.97 | 0.77 | 1.21 | 0.78 | 0.90 | 0.77 | 1.05 | 0.19 |
| Lag 0-1 | 1.67 | 0.73 | 3.83 | 0.22 | 0.96 | 0.73 | 1.26 | 0.78 | 0.51 | 0.30 | 0.86 | 0.01 | 0.94 | 0.75 | 1.16 | 0.55 |
| Lag 0-2 | 1.28 | 0.57 | 2.88 | 0.55 | 0.92 | 0.66 | 1.28 | 0.63 | 0.52 | 0.28 | 0.97 | 0.04 | 0.92 | 0.73 | 1.17 | 0.49 |
| Lag 0-3 | 0.95 | 0.37 | 2.45 | 0.91 | 0.83 | 0.55 | 1.26 | 0.38 | 0.72 | 0.35 | 1.49 | 0.38 | 0.88 | 0.68 | 1.14 | 0.34 |
| Lag 0-4 | 0.57 | 0.18 | 1.81 | 0.34 | 0.80 | 0.48 | 1.35 | 0.41 | 0.81 | 0.44 | 1.51 | 0.51 | 0.83 | 0.62 | 1.10 | 0.19 |
| Lag 0-5 | 0.57 | 0.15 | 2.24 | 0.42 | 0.66 | 0.32 | 1.34 | 0.25 | 0.82 | 0.45 | 1.49 | 0.51 | 0.76 | 0.56 | 1.05 | 0.10 |

Notes:

(a) Results for 2002 were from negative binomial regression models due to overdispersion; others were from Poisson regression models. Covariates adjusted in the Poisson regression models included apparent temperature, week, weekend, and holiday, in single-year analyses, and year, in multi-year analyses.

(b) RR was calculated for a 10 µg/m3 increase in PM_2.5_ concentrations.

**Table S8. Multivariate Regression Analyses^a^ of Daily Respiratory Mortality and PM_2.5_ in New York City between 2001-2003**

| **PM_2.5_ Lags** | **2001** | | | | **2002** | | | | **2003** | | | | **2001-2003 Combined** | | | |
| --- | --- | --- | --- | --- | --- | --- | --- | --- | --- | --- | --- | --- | --- | --- | --- | --- |
|  | **RR^b^** | **95% CI** | | **P** | **RR** | **95% CI** | | **P** | **RR** | **95% CI** | | **P** | **RR** | **95% CI** | | **P** |
| Lag 0 | 0.98 | 0.78 | 1.25 | 0.89 | 0.99 | 0.92 | 1.08 | 0.88 | 1.10 | 0.84 | 1.44 | 0.48 | 1.00 | 0.94 | 1.08 | 0.92 |
| Lag 1 | 0.95 | 0.82 | 1.10 | 0.47 | 1.06 | 0.98 | 1.14 | 0.15 | 0.97 | 0.80 | 1.16 | 0.72 | 1.02 | 0.96 | 1.08 | 0.60 |
| Lag 2 | 1.03 | 0.90 | 1.19 | 0.62 | 1.06 | 0.99 | 1.14 | 0.10 | 1.02 | 0.87 | 1.19 | 0.79 | 1.02 | 0.97 | 1.08 | 0.47 |
| Lag 3 | 1.04 | 0.94 | 1.16 | 0.43 | 1.00 | 0.93 | 1.08 | 0.93 | 1.03 | 0.91 | 1.16 | 0.64 | 1.01 | 0.96 | 1.07 | 0.66 |
| Lag 4 | 1.02 | 0.90 | 1.16 | 0.73 | 0.90 | 0.83 | 0.98 | 0.01 | 0.96 | 0.87 | 1.06 | 0.41 | 0.96 | 0.91 | 1.02 | 0.16 |
| Lag 5 | 0.99 | 0.90 | 1.09 | 0.77 | 0.95 | 0.88 | 1.04 | 0.27 | 1.06 | 0.96 | 1.18 | 0.24 | 1.00 | 0.94 | 1.05 | 0.88 |
| Lag 0-1 | 0.92 | 0.72 | 1.17 | 0.50 | 1.03 | 0.94 | 1.13 | 0.48 | 1.04 | 0.66 | 1.62 | 0.87 | 1.02 | 0.94 | 1.10 | 0.68 |
| Lag 0-2 | 0.98 | 0.76 | 1.26 | 0.87 | 1.07 | 0.97 | 1.19 | 0.18 | 1.07 | 0.72 | 1.60 | 0.74 | 1.03 | 0.95 | 1.12 | 0.50 |
| Lag 0-3 | 1.05 | 0.80 | 1.39 | 0.72 | 1.07 | 0.95 | 1.21 | 0.26 | 1.12 | 0.77 | 1.63 | 0.55 | 1.04 | 0.94 | 1.14 | 0.46 |
| Lag 0-4 | 1.07 | 0.80 | 1.43 | 0.64 | 1.03 | 0.89 | 1.19 | 0.74 | 0.98 | 0.71 | 1.34 | 0.89 | 1.01 | 0.90 | 1.12 | 0.90 |
| Lag 0-5 | 1.04 | 0.77 | 1.41 | 0.80 | 1.00 | 0.83 | 1.20 | 0.97 | 1.07 | 0.79 | 1.44 | 0.67 | 1.00 | 0.89 | 1.13 | 0.95 |

Notes:

(a) Covariates adjusted in the Poisson regression models included apparent temperature, week, weekend, and holiday, in single-year analyses, and year, in multi-year analyses.

(b) RR was calculated for a 10 µg/m3 increase in PM_2.5_ concentrations.

**Table S9. Multivariate Regression Analyses^a^ of Daily Respiratory Mortality and PM_2.5_ in Greater Boston and New York City between 2001-2003**

| **PM_2.5_ Lags** | **2001** | | | | **2002** | | | | **2003** | | | | **2001-2003 Combined** | | | |
| --- | --- | --- | --- | --- | --- | --- | --- | --- | --- | --- | --- | --- | --- | --- | --- | --- |
|  | **RR^b^** | **95% CI** | | **P** | **RR** | **95% CI** | | **P** | **RR** | **95% CI** | | **P** | **RR** | **95% CI** | | **P** |
| Lag 0 | 1.06 | 0.83 | 1.34 | 0.65 | 0.99 | 0.92 | 1.07 | 0.81 | 1.05 | 0.83 | 1.34 | 0.68 | 1.00 | 0.94 | 1.06 | 0.97 |
| Lag 1 | 0.98 | 0.83 | 1.15 | 0.79 | 1.05 | 0.97 | 1.13 | 0.23 | 0.93 | 0.77 | 1.13 | 0.46 | 1.01 | 0.95 | 1.07 | 0.76 |
| Lag 2 | 0.99 | 0.86 | 1.14 | 0.90 | 1.05 | 0.98 | 1.13 | 0.18 | 1.02 | 0.86 | 1.20 | 0.84 | 1.01 | 0.96 | 1.07 | 0.66 |
| Lag 3 | 1.01 | 0.90 | 1.13 | 0.90 | 1.00 | 0.92 | 1.08 | 0.96 | 1.05 | 0.92 | 1.19 | 0.47 | 1.00 | 0.95 | 1.05 | 0.93 |
| Lag 4 | 0.97 | 0.84 | 1.11 | 0.63 | 0.92 | 0.85 | 1.01 | 0.08 | 0.97 | 0.87 | 1.08 | 0.59 | 0.95 | 0.90 | 1.01 | 0.08 |
| Lag 5 | 0.98 | 0.88 | 1.10 | 0.77 | 0.95 | 0.87 | 1.03 | 0.22 | 1.06 | 0.94 | 1.19 | 0.34 | 0.98 | 0.93 | 1.03 | 0.49 |
| Lag 0-1 | 1.00 | 0.77 | 1.30 | 0.98 | 1.03 | 0.94 | 1.12 | 0.58 | 0.91 | 0.59 | 1.39 | 0.66 | 1.01 | 0.93 | 1.09 | 0.86 |
| Lag 0-2 | 0.99 | 0.76 | 1.29 | 0.95 | 1.06 | 0.95 | 1.18 | 0.29 | 0.98 | 0.65 | 1.47 | 0.91 | 1.02 | 0.94 | 1.10 | 0.72 |
| Lag 0-3 | 1.00 | 0.75 | 1.35 | 0.98 | 1.06 | 0.94 | 1.20 | 0.37 | 1.10 | 0.74 | 1.64 | 0.64 | 1.02 | 0.93 | 1.11 | 0.74 |
| Lag 0-4 | 0.97 | 0.71 | 1.32 | 0.84 | 1.02 | 0.88 | 1.18 | 0.83 | 1.00 | 0.72 | 1.38 | 0.98 | 0.98 | 0.89 | 1.08 | 0.70 |
| Lag 0-5 | 0.95 | 0.68 | 1.31 | 0.75 | 0.98 | 0.81 | 1.18 | 0.82 | 1.07 | 0.78 | 1.48 | 0.67 | 0.97 | 0.86 | 1.08 | 0.54 |

Notes:

(a) Random-effects models were used to combine the Poisson regression analyses between Greater Boston and New York City. Covariates adjusted in the Poisson regression models included apparent temperature, week, weekend, and holiday, in single-year analyses, and year, in multi-year analyses.

(b) RR was calculated for a 10 µg/m3 increase in PM_2.5_ concentrations.
